# Diabetic retinopathy is associated with the presence and burden of subclinical carotid atherosclerosis in type 1 diabetes

**DOI:** 10.1186/s12933-018-0706-z

**Published:** 2018-05-04

**Authors:** Marc Carbonell, Esmeralda Castelblanco, Xavier Valldeperas, Àngels Betriu, Alícia Traveset, Minerva Granado-Casas, Marta Hernández, Federico Vázquez, Mariona Martín, Esther Rubinat, Albert Lecube, Josep Franch-Nadal, Elvira Fernández, Manel Puig-Domingo, Angelo Avogaro, Núria Alonso, Dídac Mauricio

**Affiliations:** 1 Department of Ophthalmology, University Hospital Germans Trias i Pujol, Badalona, Spain; 2 Department of Endocrinology and Nutrition, University Hospital and Health Science Research Institute Germans Trias i Pujol, Carretera Canyet S/N, 08916 Badalona, Spain; 3 Center for Biomedical Research on Diabetes and Associated Metabolic Diseases (CIBERDEM), Barcelona, Spain; 4 Biomedical Research Institute of Lleida, Lleida, Spain; 5 Department of Endocrinology and Nutrition, University Hospital Arnau de Vilanova, Lleida, Spain; 6 Department of Ophthalmology, University Hospital Arnau de Vilanova, Lleida, Spain; 7 Department of Medicine, Barcelona Autonomous University (UAB), Barcelona, Spain; 8 Department of Surgery, Barcelona Autonomous University (UAB), Barcelona, Spain; 9 Primary Health Care Center Raval Sud, Gerència d’Atenció Primaria, Institut Català de la Salut, Barcelona, Spain; 10 Department of Medicine, University of Padova, Padua, Italy

**Keywords:** Type 1 diabetes, Retinopathy, Subclinical carotid atherosclerosis

## Abstract

**Background:**

Cardiovascular (CV) disease due to atherosclerosis is a major cause of morbidity and mortality in adult patients with diabetes, either type 1 or type 2 diabetes. The aim of the study was to assess the association of the frequency and the burden of subclinical carotid atherosclerotic disease in patients with type 1 diabetes according to the presence and severity of diabetic retinopathy (DR).

**Methods:**

A cross-sectional study was conducted in 340 patients with type 1 diabetes (41.5% with DR), and in 304 non-diabetic individuals. All participants were free from previous CV disease and chronic kidney disease (CKD). B-mode carotid ultrasound imaging was performed in all the study subjects. Patients with type 1 diabetes underwent a full eye examination, and DR patients were divided into two groups: mild disease and advanced disease.

**Results:**

In the group of patients with type 1 diabetes, the percentage of patients with carotid plaques was higher in those with DR compared with those without DR (44.7% vs. 24.1%, p < 0.001). Patients with DR also presented a higher incidence of ≥ 2 carotid plaques (25.5% vs. 11.1%, p < 0.001). Apart from other traditional cardiovascular risk factors, the presence of advanced stages of DR was independently associated with the presence (p = 0.044) and the burden (≥ 2 carotid plaques; p = 0.009) of subclinical carotid atherosclerosis.

**Conclusions:**

In patients with type 1 diabetes without previous CV disease or established CKD, the presence of advanced stages of DR is associated with a higher atherosclerotic burden in the carotid arteries. The presence of DR identifies patients at risk for carotid atherosclerotic disease.

**Electronic supplementary material:**

The online version of this article (10.1186/s12933-018-0706-z) contains supplementary material, which is available to authorized users.

## Background

Cardiovascular (CV) disease due to atherosclerosis is a major cause of morbidity and mortality in adult patients with diabetes, either type 1 or type 2 diabetes. Among the risk factors associated with the excess of cardiovascular risk in patients with type 1 diabetes are age, proteinuria, decreased glomerular filtration rate, hypertension, obesity, poor glycemic control, lipid levels, smoking and diabetes duration [1]. Even in patients with optimal glycemic control, the risk of death from CV causes is still more than twice that of non-diabetic individuals [2]. Unlike patients with type 2 diabetes, those with type 1 diabetes generally do not have excess rates of obesity, hypertension, or hypercholesterolemia compared with the general population [3]. Thus, the underlying mechanisms responsible for the increased risk of death from any cause and of death from CV causes among patients with type 1 diabetes who have good glycemic control are not fully understood. In recent years, a current line of thought has been that there is a “common soil” for the development of micro- and macrovascular complications of diabetes associated with common pathogenic pathways in both types of complications [4]. Diabetic retinopathy (DR) is a common and specific microvascular complication of diabetes that is identified in one-third of people with type 1 diabetes [5]. Glycemic control is the main factor involved in appearance and progression of DR in these patients. Interestingly, the presence of morphological abnormalities in retinal microvasculature is even observed in childhood type 1 diabetic patients with short-term poor glycemic control [6].

It has been shown in several studies that DR is an independent predictor of CV events and all-cause mortality in both patients with type 1 and type 2 diabetes [7]. Multiple potential mechanisms have been suggested to be involved in the pathophysiological link between diabetic micro- and macroangiopathy. These include oxidative and glycemic stress, chronic low-grade inflammation and impaired vascular tissue repair mechanisms [4]. More recently, diabetic microangiopathy of the artery wall microvessels, named vasa vasorum, has also been reported in patients with type 2 diabetes [8]. It is interesting to note that the main stimuli that drive diabetic microangiopathy angiogenesis are hypoxia and ischemia similar to what has been described in the retinal vascular changes that occur in DR [5]. Interestingly, the severity of retinopathy has been shown to be associated with the risk of incidental CV events in patients with type 2 diabetes in the action to control cardiovascular risk in diabetes (ACCORD) eye study, as this risk increased from 1.5 in people with mild nonproliferative retinopathy to 2.4 in people with severe retinopathy [9]. In patients with type 1 diabetes asymptomatic for coronary artery disease, severe forms of DR have been reported to be associated with the presence of coronary artery calcification [10], and also with increased carotid intima-media thickness [11].

On the other hand, established chronic kidney disease (CKD) is one of the main drivers of increased CV morbidity and mortality in patients with type 1 diabetes. Specifically, in patients with type 1 diabetes, the presence of albuminuric [12] and nonalbuminuric [13] CKD is associated with a higher risk of CV disease. In previous studies showing an increased CV risk in patients with DR, the inclusion of patients with CKD may have influenced the findings as DR is usually closely associated with diabetic nephropathy. Therefore, any studies considering the association of DR with CV disease should take this issue into consideration.

Carotid intima-media thickness (IMT) and the presence of carotid plaques are considered to be surrogate measures of atherosclerosis [14]. Both conditions are associated with CV risk factors [15], predict CV events independently of risk factors [16], and help to stratify patients into different risk categories [17]. However, it should be mentioned that the presence [18] and burden [19] of atherosclerotic plaque is a more powerful predictor of CV risk compared with IMT alone.

Both IMT and the frequency of plaques have been reported to be increased in children, adolescents and adults with type 1 diabetes compared with age- and sex-matched healthy control subjects [1], although data regarding atherosclerotic plaques in patients with type 1 diabetes are very scarce. A paucity of data exists on whether DR and subclinical atherosclerotic disease in the carotid artery territory in patients with type 1 diabetes are associated. Carotid plaque is recognized as an independent risk factor for ischemic stroke [20] with a risk that is markedly increased both in patients with type 1 and type 2 diabetes [21]. In patients with type 1 diabetes, this risk has been described to be associated with the presence of diabetic nephropathy and severe diabetic retinopathy [22]. In patients with type 2 diabetes, ours and other groups have previously described that DR is an independent risk factor associated with the presence of carotid plaques in subjects without previous CV disease [23, 24]. In these patients, it has recently been described that carotid atherosclerosis is also an independent risk factor for stroke [25].

Therefore, we hypothesized that DR is associated with the presence of carotid plaques in subjects with type 1 diabetes without previous CV disease and established chronic kidney disease. In the present study, we sought to assess the association of the frequency and burden of subclinical carotid atherosclerotic disease in patients with type 1 diabetes according to presence and severity of DR. As a secondary outcome, we also evaluated the differences in the presence and burden of subclinical carotid atherosclerosis compared with non-diabetic subjects.

## Methods

### Subjects

This study was designed as a cross-sectional study in subjects with type 1 diabetes recruited from the diabetic outpatient clinics at two university hospitals in the North-Western region of Spain (Catalonia). All potential participants were identified from the electronic clinical records from the two participating institutions that belong to the same health care organization.

The inclusion criteria for type 1 diabetic subjects were as follows: age > 18 years; diabetes for at least 1 year; normal renal function [estimated glomerular filtration rate (eGFR > 60 mL/min)]; no previous cardiovascular (CV) disease defined as any form of clinical coronary heart disease, stroke or peripheral vascular disease; and any form of diabetic foot disease. We excluded patients with a urine albumin excretion ratio > 300 mg/g and any conditions that precluded a proper full eye examination. A subject was considered to have previous hypertension or dyslipidaemia if he/she was taking medication for the given condition. The selection of non-diabetic control participants was based on the same criteria, except for the criteria that concerned the diabetic specific microvascular complications (retinopathy and albuminuria). Additionally, subjects in the control group had fasting glucose and HbA1c values below 100 mg/dL and 5.7% HbA1c, respectively.

### Clinical assessment

For each subject, the age, sex, weight, height, body mass index and waist circumference were obtained by standardized methods. Serum and spot urine samples were collected in the fasting state, and all serum and urine tests were performed using standard laboratory methods as previously described [23].

### Ophthalmic examination

In subjects with type 1 diabetes, an experienced ophthalmologist performed a complete eye evaluation and defined DR according to the ETDRS classification in five stages: (1) no apparent retinopathy, (2) mild non-proliferative retinopathy (NPDR) (defined by the presence of microaneurysms), (3) moderate NPDR (presence of microaneurysms, intraretinal hemorrhages or venous beading that did not reach the severity of the next stage), (4) severe NPDR (more than 20 intraretinal hemorrhages in four quadrants, venous beading in two quadrants or intraretinal microvascular abnormalities in one quadrant), and (5) proliferative diabetic retinopathy (PRD) defined by the presence of disc/retina/angle/iris neovascularization, vitreous hemorrhage or tractional retinal detachment [26]. For the purpose of the analysis, patients with DR were classified into two groups according to the severity of diabetic retinopathy: mild diabetic retinopathy and advanced DR (stages 3, 4 and 5 of the ETDRS classification).

### Carotid ultrasound imaging

All the study participants underwent the same carotid ultrasound examination. Carotid ultrasonography imaging was performed using a LOGIQ^®^ E9-General Electric (Wauwatosa, WI 53226, USA) equipped with a 15 MHz linear array probe or a Sequoia 512, Siemens, (North Rhine, Westphalia, Germany) equipped with a 15 MHz linear array probe. All measures and ultrasound studies were assessed by the same researcher at each participating hospital. The detailed carotid ultrasound protocol performed to evaluate the presence of carotid plaques has been previously described [23]. Plaques were identified using B-mode and color Doppler examinations in both the longitudinal and transverse planes to consider circumferential asymmetry and were defined as a “focal structure that encroaches into the arterial lumen of at least 0.5 mm or 50% of the surrounding carotid IMT value or demonstrates a thickness of 1.5 mm, as measured from the media-adventitia interference to the intima-lumen surface” according to the Mannheim consensus [27]. The arterial territories explored included the left and right carotid arteries; for each of them, the common and internal carotid territories and their bifurcation were examined.

The Local Ethics Committee of both participating centers approved the protocol, and all the participants signed informed consent forms.

### Statistical analysis

The descriptive statistics of the mean (standard deviation) or median [interquartile range] were estimated for quantitative variables with a normal or non-normal distribution, respectively. For the qualitative variables, absolute and relative frequencies were used. The normal distribution was analyzed by the Shapiro–Wilks test. The differences between groups were assessed by Student’s test, analysis of variance (ANOVA) or Mann–Whitney test, and Kruskal–Wallis test depending on the distributions of the quantitative variables. The significance of the differences in qualitative variables was assessed by Chi squared test or Fisher’s exact test. Logistic regression and multinomial logistic regression models were performed to study the prevalence and burden of subclinical carotid atherosclerotic disease and its association with DR. All variables of the bivariate analysis with a p < 0.2 were used. Only the main effects with a significant contribution to the final model according to the likelihood ratio test were included in the final model. For logistic regression model calibration and discrimination, the Homer–Lemeshow test and the area under the ROC curve were used. The R statistical software, version 3.3.1, was used for all the analyses, using a significance level of 0.05.

### Sample size

Based on a predefined prevalence of DR in patients with type 1 diabetes of 33%, we estimated a sample size of 309 patients with type 1 diabetes as being sufficient to detect a difference in the frequency of carotid atherosclerotic plaques of 15% between patients with type 1 diabetes with and without DR, assuming frequencies of plaques of 35 and 20% in patients with and without DR, respectively. This assumption yielded an overall frequency of plaques of 25% in this sample of subjects with type 1 diabetes. A comparison with a group with the same number of non-diabetic subjects would then allow the detection of a difference of 9% between them and type 1 diabetes.

## Results

From the initial number of type 1 diabetic patients recruited (n = 397) in the study, five patients were excluded for presenting an eGFR < 60 mL/min, two patients were excluded for presenting a urine albumin/creatinine ratio > 300 mg/g, three patients were excluded for presenting cardiovascular disease and 11 patients were excluded for presenting with one or more ophthalmic exclusion criteria. Additionally, six patients did not attend the carotid ultrasound examination, and 30 patients did not complete all the determinations of the main study outcomes. Therefore, 340 patients with type 1 diabetes were finally enrolled in the study. We enrolled more subjects in the type 1 diabetes group as we allowed recruitment in the two centers until the control group was completed. A total of 332 individuals were contacted for inclusion in the control group. From this initial number, nine individuals did not accept participation, and 19 were excluded because of the presence of exclusion criteria or for not completing all the study procedures. Therefore, from the expected size of the group of non-diabetic individuals (n = 309), a total of 304 individuals were recruited.

### Clinical variables

The results of the study variables for each study group are shown in Table 1. Among the 340 study patients with type 1 diabetes, 141 (41.5%) had DR. The patients with DR were older, had a higher prevalence of hypertension and dyslipidemia and a longer diabetes duration. These patients also exhibited higher systolic blood pressure, pulse pressure, BMI, waist circumference, plasma triglycerides and HbA1c compared with those without DR (Table 1). The distribution of the stages of DR in the 141 patients with DR was as follows: 89 patients had mild DR, 18 patients had moderate DR, 15 patients had severe DR, and 19 patients had proliferative DR. For further analysis, we classified the status of retinopathy into non-DR (n = 199, 58.5%), mild DR (n = 89, 26.2%), and advanced DR (including moderate, severe and proliferative disease) (n = 52, 15.3%). The clinical characteristics of these 3 groups of patients are shown in Table 2.

**Table 1:** Clinical characteristics of the study groups

	Control	Type 1 diabetes			p. control vs. type 1 diabetes	p. no DR vs. DR
		All	No retinopathy	Retinopathy		
	N = 307	N = 340	N = 199	N = 141		
Age, years	44.0 [37.0; 51.0]	45.0 [37.0; 53.0]	43.4 (11.1)	48.6 (12.1)	0.164	< 0.001
Sex, men	139 (45.7%)	155 (45.6%)	89 (44.7%)	66 (46.8%)	1	0.787
Non-caucasian	9 (2.96%)	4 (1.18%)	3 (1.51%)	1 (0.71%)	0.185	0.645
Current or former smoker	167 (55.3%)	174 (51.3%)	97 (49.0%)	77 (54.6%)	0.354	0.363
Dyslipidemia	39 (12.8%)	144 (42.4%)	72 (36.2%)	72 (51.1%)	< 0.001	0.009
Hypertension	26 (8.55%)	88 (25.9%)	36 (18.1%)	52 (36.9%)	< 0.001	< 0.001
Systolic BP, mmHg	120 [112; 129]	129 [114; 139]	127 [112; 135]	132 [119; 145]	< 0.001	< 0.001
Diastolic BP, mmHg	76.0 [70.0; 81.0]	75.0 [68.0; 80.0]	74.5 (10.1)	73.7 (9.98)	0.009	0.447
Pulse pressure, mmHg	44.0 [38.0; 51.8]	52.0 [42.5; 62.0]	50.0 [40.0; 58.5]	56.0 [46.8; 70.0]	< 0.001	< 0.001
BMI kg/m^2^	25.2 [23.2; 28.4]	25.6 [22.8; 28.4]	25.0 [22.5; 27.4]	26.3 [23.5; 29.4]	0.827	0.004
Waist circumference, cm	93.0 [84.2; 100]	88.0 [80.0; 98.5]	87.0 [79.0; 95.0]	90.0 [82.0; 102]	< 0.001	0.003
HbA1c, %	5.40 [5.20; 5.60]	7.50 [7.00; 8.10]	7.40 [6.80; 7.90]	7.60 [7.20; 8.40]	<0.001	< 0.001
HbA1c, mmol/mol	36.0 [33.0; 38.0]	58.0 [53.0; 65.0]	57.0 [51.0; 63.0]	60.0 [55.0; 68.0]	< 0.001	< 0.001
Total-c, mg/dL	192 [170; 220]	176 [160; 200]	175 [161; 200]	177 [156; 201]	< 0.001	0.903
HDL, mg/dL	56.5 [47.0; 68.0]	62.0 [53.0; 74.0]	63.0 [54.0; 74.0]	60.5 [55.0; 72.8]	< 0.001	0.197
LDL, mg/dL	116 [94.8; 138]	99.4 [83.0; 116]	99.4 [84.6; 116]	99.5 [79.9; 117]	< 0.001	0.684
Triglycerides, mg/dL	85.0 [63.0; 122]	68.0 [53.0; 86.0]	65.0 [53.0; 83.5]	70.0 [55.0; 89.0]	< 0.001	0.071
Creatinine, mg/dL	0.78 [0.68; 0.90]	0.76 [0.65; 0.87]	0.77 [0.66; 0.88]	0.76 [0.65; 0.87]	0.044	0.890
Urine albumin/creatinine ratio, mg/g	–	3.96 [2.08; 6.84]	3.58 [1.82; 5.95]	4.70 [2.76; 8.34]	–	0.030
Diabetes duration, years	–	20.0 [14.0; 29.0]	16.0 [11.0; 22.0]	27.0 [20.0; 33.0]	–	< 0.001

Variables are expressed as median and interquartile range, unless otherwise specified

*BMI* body mass index, *BP* blood pressure, *HDL* high-density lipoprotein cholesterol, *LDL* low-density lipoprotein cholesterol, *Total-c* total cholesterol

**Table 2:** Clinical characteristics of patients with type 1 diabetes according to the presence and severity of DR

	No DR	Mild DR	> Mild DR	p overall
	N = 199	N = 89	N = 52	
Age, years	43.4 (11.1)	47.1 (12.2)	51.1 (11.6)	< 0.001
Sex, men	89 (44.7%)	41 (46.1%)	27 (51.9%)	0.906
Non-caucasian	3 (1.51%)	1 (1.12%)	0 (0.00%)	1
Current or former smoker	97 (49.0%)	49 (55.1%)	28 (53.8%)	0.588
Antiplatelet	46 (23.1%)	23 (25.8%)	23 (44.2%)	0.009
Dyslipidemia	72 (36.2%)	41 (46.1%)	31 (59.6%)	0.007
Hypertension	36 (18.1%)	25 (28.1%)	27 (51.9%)	< 0.001
Systolic BP, mmHg	127 [112; 135]	130 [119; 140]	136 [116; 150]	0.001
Diastolic BP, mmHg	74.5 (10.1)	74.4 (8.92)	72.4 (11.6)	0.395
Pulse pressure, mmHg	50.0 [40.0; 58.5]	53.0 [45.0; 66.0]	62.0 [50.5; 75.0]	< 0.001
BMI, kg/m^2^	25.0 [22.5; 27.4]	26.2 [23.6; 29.4]	26.3 [23.3; 30.2]	0.013
Waist circumference, cm	87.0 [79.0; 95.0]	90.0 [82.0; 100]	91.5 [82.0; 103]	0.009
HbA1c, %	7.40 [6.80; 7.90]	7.50 [7.10; 8.20]	7.90 [7.30; 8.60]	< 0.001
HbA1c, mmol/mol	57.0 [51.0; 63.0]	58.0 [54.0; 66.0]	63.0 [56.0; 70.0]	< 0.001
Total-c, mg/dL	175 [161; 200]	177 [154; 200]	184 [157; 201]	0.989
HDL, mg/dL	63.0 [54.0; 74.0]	60.5 [51.8; 71.0]	60.5 [50.8; 76.0]	0.422
LDL, mg/dL	99.4 [84.6; 116]	98.0 [81.8; 121]	105 [78.1; 115]	0.913
Triglycerides, mg/dL	65.0 [53.0; 83.5]	69.0 [53.0; 93.0]	74.0 [56.8; 88.0]	0.166
Creatinine, mg/dL	0.77 [0.66; 0.88]	0.75 [0.64; 0.85]	0.78 [0.68; 0.92]	0.340
Urine albumin/creatinine ratio mg/g	3.58 [1.82; 5.95]	4.00 [1.98; 5.62]	5.71 [3.12; 16.3]	0.001
Diabetes duration	16.0 [11.0; 22.0]	25.0 [18.0; 30.0]	30.5 [22.0; 38.0]	< 0.001

Variables are expressed as median and interquartile range, unless otherwise specified

*BMI* body mass index, *BP* blood pressure, *HDL* high-density lipoprotein cholesterol, *LDL* low-density lipoprotein cholesterol, *Total-c* total cholesterol

### Ultrasound examination

**Fig. 1:**
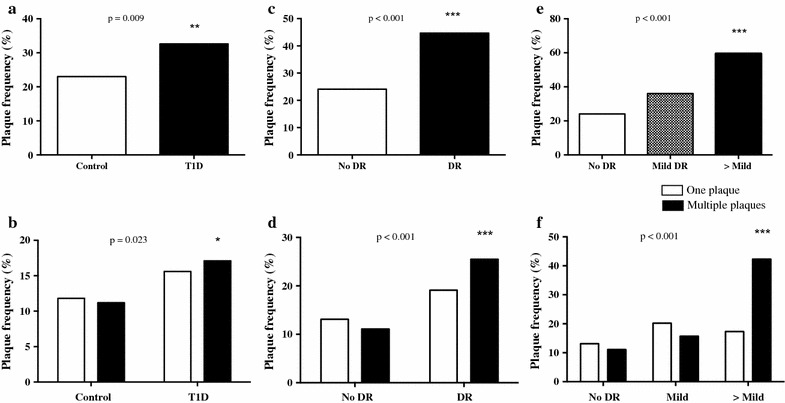
Percentage of patients with the presence of one atherosclerotic plaque (**a**) or multiple plaques (**b**) in the control group and in patients with type 1 diabetes. Percentage of patients with type 1 diabetes with the presence of one atherosclerotic plaque or multiple plaques according to the presence or absence of DR (**c**, **d**). Percentage of patients with type 1 diabetes with the presence of one atherosclerotic plaque (**e**) or multiple plaques (**f**) according to the degree of DR

The percentage of patients with carotid plaques was higher in patients with type 1 diabetes compared with control subjects (32.6% vs. 23%, p = 0.009). The patients with type 1 diabetes also presented with a higher percentage of ≥ 2 carotid plaques (17.1% vs. 11.2%, p = 0.023) compared with non-diabetic subjects. In the group of patients with type 1 diabetes, the percentage of patients with carotid plaques was higher in those with DR compared with those without DR (44.7% vs. 24.1%, p < 0.001). The patients with DR also presented a higher percentage of ≥ 2 carotid plaques (25.5% vs. 11.1%, p < 0.001) compared to those without DR (Fig. 1).

### Frequency and burden of atherosclerotic plaques in patients with type 1 diabetes

**Fig. 2:**
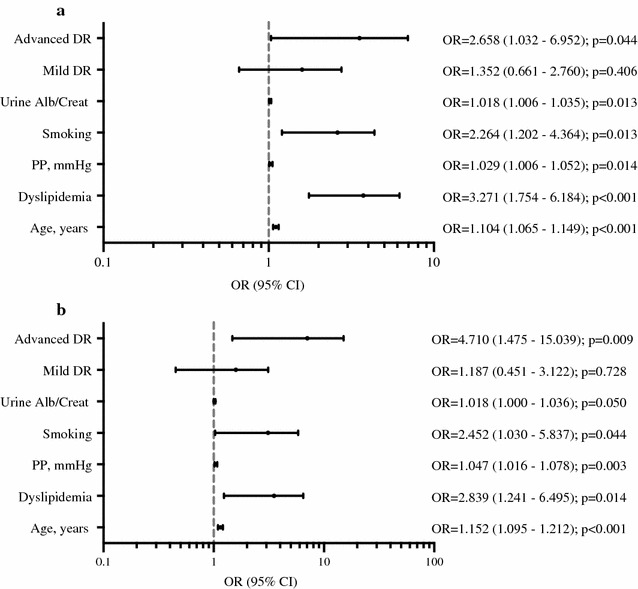
Multivariate logistic regression models: **a** model for the presence or absence of carotid plaque in patients with type 1 diabetes. **b** Model for absence vs presence of multiple (≥ 2) plaques in patients with type 1 diabetes. Models adjusted by sex, age, diabetes duration, smoking, diastolic BP, dyslipidemia, diabetic retinopathy, urine albumin/creatinine ratio, BMI, pulse pressure and LDL. Smoking refers to current and former smokers

In the group of patients with type 1 diabetes, as the frequency of subclinical carotid atherosclerosis was mainly at the expense of patients with advanced DR, we performed a multiple regression analysis that used the group of patients without DR as the reference group. This analysis revealed that the variables associated with the presence of carotid plaques were age (p < 0.001), advanced DR (p = 0.044), smoking (p = 0.013), dyslipidemia (p < 0.001), pulse pressure (p = 0.014), and albumin creatinine ratio (p = 0.013) (Fig. 2a).

Next, we performed the multivariate logistic regression analysis for the presence of one plaque or multiple (≥ 2) plaques (Additional file 1: Table S1). The variables associated with multiple plaques were age, smoking, dyslipidemia, advanced stages of retinopathy, pulse pressure and albumin creatinine ratio (Fig. 2b).

We also performed a similar multivariate logistic regression analysis for the presence and absence of DR as a microvascular complication of atherosclerosis. In this analysis, we did not find a significant contribution of retinopathy (p = 0.140) for the presence of subclinical carotid atherosclerosis (Additional file 1: Table S2).

### Frequency and burden of atherosclerotic plaques in the whole study group

To analyze the variables associated with the presence and burden of atherosclerotic plaques in the whole group (type 1 diabetes and non-diabetic participants), we performed a logistic regression analysis. We first analyzed the variables associated with the presence of atherosclerotic plaques. In this model, we found an interaction of diabetes with smoking. The variables associated with presence of any plaque were age (p < 0.001), dyslipidemia (p < 0.001), pulse pressure (p < 0.001) and smoking if type 1 diabetes (p = 0.003) (Additional file 1: Table S3).

Next, we performed the multivariate logistic regression analysis for the presence of one plaque or multiple (≥ 2) plaques (Additional file 1: Table S3). There were several variables that were consistently associated with the presence of one or multiples plaques: age, dyslipidemia, pulse pressure and smoking if type 1 diabetes. In contrast, only one variable was associated with the presence of multiple plaques, the sex.

## Discussion

In the present study, that included a large sample of patients with type 1 diabetes and an age- and sex-matched control group of participants without diabetes, the frequency of carotid plaques was significantly higher in patients with type 1 diabetes. Interestingly, apart from other well-known cardiovascular risk factors, the presence of advanced DR was an independent predictor of the presence of carotid plaques and of the atherosclerotic burden in type 1 diabetes.

The natural history of atherosclerosis involves a protracted subclinical phase, with the atherosclerotic disease often being detected only at an advanced stage or after a CV event. Non-invasive methods, such as arterial ultrasonography, are used to detect and quantify atherosclerosis and have shown to be a useful screening test for the prediction of future CV events. It is now generally accepted that presence and burden of carotid plaques compared with that of carotid IMT has a higher diagnostic accuracy for the prediction of future CV events [17, 18]. Several studies showed that patients with type 1 diabetes had a significantly increased carotid IMT compared with age- and sex-matched non-diabetic subjects [28]. Less information is available regarding the presence of atherosclerotic plaques in patients with type 1 diabetes, as well as its association with the presence of DR. In a study by Distiller et al. in 148 patients with type 1 diabetes (mean age 48 years, and mean diabetes duration of 26 years), the prevalence of plaques was 18.9%. In this study, DR was reported to be independently associated with the presence of plaques [29]. Additionally, Ogawa et al. [30] described a plaque frequency of 28.8% in a group of 73 patients with type 1 diabetes (mean age 38 years) with early-onset long-duration type 1 diabetes. This study reported an association between proliferative DR with a maximal carotid IMT of the whole carotid artery. Patients with proliferative DR from that study also showed a non-significant higher frequency of plaques compared with those without this condition (42.9% vs. 23.1%). In the current study, the presence of plaques was significantly higher in patients with type 1 diabetes compared with non-diabetic subjects. Moreover, a higher proportion  of patients with type 1 diabetes presented with ≥ 2 carotid plaques compared with controls. The factors independently associated with carotid atherosclerotic plaques in patients with type 1 diabetes were different depending on whether only one plaque or ≥ 2 carotid plaques were present. In both cases, well-known cardiovascular risk factors and, more importantly, advanced stages of DR were independently associated with subclinical atherosclerosis. Interestingly, in a recent study, we have reported that the frequency of preclinical carotid atherosclerosis in patients with latent autoimmune diabetes of the adults (LADA) is similar, or even greater in those with longer disease duration, to subjects of similar age with classic type 1 diabetes and type 2 diabetes [31]. In LADA, preclinical carotid atherosclerosis was also associated with classical risk factors like hypertension and tobacco exposure.

The data related to the presence and extent of carotid plaques in patients with type 2 diabetes are more abundant [32]. In these patients, a high prevalence of carotid plaque presence and burden compared with healthy subjects has been described [33]. In patients with type 2 diabetes, ours and other groups have previously described that DR is an independent risk factor associated with the presence of carotid plaques in subjects without previous cardiovascular disease [23, 24]. Specifically, the proportion of carotid plaques in type 2 diabetes patients with DR was higher than in type 2 diabetes patients without DR; additionally, patients with DR had a higher burden of atherosclerosis (≥ 2 carotid plaques) [23]. Further, patients with type 2 diabetes and advanced stages of retinopathy have stenotic atherosclerotic plaques more frequently than patients with mild retinopathy [24].

Another proposed measure to evaluate the presence and progression of microvascular complications in patients with type 1 diabetes is the analysis of advanced glycation end-products (AGEs). In these patients, it has been described that accumulation of AGEs in the skin is independently associated with progression of retinopathy as well as with presence and progression of subclinical cardiovascular disease (i.e. the severity of coronary artery calcification and the IMT carotid progression) [34, 35]. Other authors have suggested factors, other than the presence and burden of atherosclerotic plaques, that may be involved in the increased cardiovascular risk in patients with type 1 diabetes, i.e. increased arterial stiffness [36, 37], and HDL dysfunction [38].

Diabetic retinopathy has been described to be associated with macrovascular disease [39], as well as with increased CV morbidity and mortality [7]. It is interesting to note that the presence of severe DR is among the independent risk factors for stroke both in patients with type 1 diabetes [7, 22] and in patients with type 2 diabetes [40]. Thus, the presence and extent of carotid atheromatous disease described in the present study in patients with type 1 diabetes and DR may be one of the factors associated with the higher risk of stroke described in patients with type 1 diabetes given that the presence of carotid plaque is an accepted independent risk factor for stroke. Lipid concentrations are strongly related to the risk of cardiovascular disease.

In patients with type 1 diabetes, it has been reported that in the context of good glycemic control HDL cholesterol is often similar or higher, and triglycerides and LDL cholesterol are often lower in type 1 diabetes in comparison with non-diabetic subjects [41]. This is consistent with the fair glycemic control observed in type 1 diabetes patients in our study. In the current study, the increased HDL concentration observed in type 1 diabetes compared with control subjects may be related to the increased waist circumference in control subjects. The latter has already been found to be associated with reduced HDL concentrations in patients with type 1 diabetes [42].

The current study has several limitations. First, the study design does not allow for the identification of a pathophysiological link between DR and atherosclerosis. Additionally, the initial sample size calculation was primarily based on the presence of DR and not on the severity of this microvascular complication; given our findings, a larger study may be warranted to confirm the current results.

## Conclusions

The present study performed in a large cohort of patients with type 1 diabetes free from CV disease shows a higher prevalence and burden of carotid atherosclerotic disease in these patients. In a similar way to what has been described in patients with type 2 diabetes, the presence of advanced stages of DR is independently associated with the presence of carotid plaques and the burden of atherosclerotic disease in patients with type 1 diabetes. Given the association described between the atherosclerotic plaque burden and the risk of future CV events in the general population, the patients with type 1 diabetes and DR may be a subgroup of patients who should be regarded at increased risk for future CV events and should be followed more closely to prevent CV disease. The follow-up of the whole cohort of our patients with type 1 diabetes may help to address these issues. The current findings should lead to an increased awareness of DR as a non-classic cardiovascular risk factor for type 1 diabetes and to its proper consideration for preventive treatment among clinicians.

## Additional file

**Additional file 1: Table S1.** Multivariate and multinomial logistic regression models of the presence/absence of atherosclerotic plaque, none vs one plaque, and none vs multiple plaques in patients with type 1 diabetes with DR as a variable introduced as stages No/Mild/>Mild. **Table S2.** Multivariate and multinomial logistic regression models of the presence/absence of atherosclerotic plaque, none vs one plaque, and none vs multiple (≥ 2) plaques in patients with type 1 diabetes with DR as a variable introduced as presence/absence. **Table S3.** Multivariate and multinomial logistic regression models of the presence/absence of atherosclerotic plaque, none vs one plaque, and none vs multiple (≥ 2) plaques in the whole study group.

## Abbreviations

CV: cardiovascular
DR: diabetic retinopathy
CKD: chronic kidney disease
IMT: intima-media thickness
eGFR: estimated glomerular filtration rate
