# Supplementary material for: Diabetic retinopathy is associated with the presence and burden of subclinical carotid atherosclerosis in type 1 diabetes
Source: Cardiovasc Diabetol. 2018 May 4;17:66. doi: 10.1186/s12933-018-0706-z (PMC5935933; doi:10.1186/s12933-018-0706-z)
Supplement: Supplementary file 1 — Additional file 1: Table S1. Multivariate and multinomial logistic regression models of the presence/absence of atherosclerotic plaque, none vs one plaque, and none vs multiple plaques in patients with type 1 diabetes with DR as a variable introduced as stages No/Mild/>Mild. Table S2. Multivariate and multinomial logistic regression models of the presence/absence of atherosclerotic plaque, none vs one plaque, and none vs multiple (≥ 2) plaques in patients with type 1 diabetes with DR as a variable introduced as presence/absence. Table S3. Multivariate and multinomial logistic regression models of the presence/absence of atherosclerotic plaque, none vs one plaque, and none vs multiple (≥ 2) plaques in the whole study group. [file 12933_2018_706_MOESM1_ESM.docx]

**Table S1.** Multivariate and multinomial logistic regression models of the presence/absence of atherosclerotic plaque, none vs one plaque, and none *vs* multiple plaques in patients with type 1 diabetes with DR as a variable introduced as stages No/Mild/>Mild.

|  | **Presence of any carotid plaques**  **Vs. No plaque *** | | | **Burden of carotid plaques †** | | | | | |
| --- | --- | --- | --- | --- | --- | --- | --- | --- | --- |
|  |  |  |  | **One plaque vs. No plaque** | | | **Multiple plaques vs. No plaque** | | |
|  | **OR** | **95% CI** | **p** | **OR** | **95% CI** | **P** | **OR** | **95% CI** | **p** |
| Sex, woman | 0.830 | 0.431 - 1.591 | 0.574 | 1.015 | 0.479 - 2.152 | 0.968 | 0.579 | 0.243 - 1.378 | 0.218 |
| Age, years | 1.104 | 1.065 - 1.149 | <0.001 | 1.068 | 1.022 - 1.116 | 0.003 | 1.152 | 1.095 - 1.212 | <0.001 |
| BMI, Kg/m2 | 1.009 | 0.934 - 1.090 | 0.819 | 1.005 | 0.920 - 1.098 | 0.911 | 1.009 | 0.912 - 1.115 | 0.869 |
| Dyslipidemia | 3.271 | 1.754 - 6.184 | <0.001 | 3.874 | 1.809 - 8.297 | 0.001 | 2.839 | 1.241 - 6.495 | 0.014 |
| LDL, mg/dL | 1.006 | 0.995 - 1.018 | 0.310 | 1.003 | 0.990 - 1.016 | 0.678 | 1.012 | 0.997 - 1.028 | 0.123 |
| DBP, mmHg | 1.000 | 0.968 - 1.032 | 0.992 | 1.016 | 0.979 - 1.054 | 0.406 | 0.978 | 0.938 - 1.019 | 0.281 |
| PP, mmHg | 1.029 | 1.006 - 1.052 | 0.014 | 1.017 | 0.991 - 1.044 | 0.205 | 1.047 | 1.016 - 1.078 | 0.003 |
| Smoking | 2.264 | 1.202 - 4.364 | 0.013 | 2.146 | 1.027 - 4.485 | 0.043 | 2.452 | 1.030 - 5.837 | 0.044 |
| Urine alb/creat | 1.018 | 1.006 - 1.035 | 0.013 | 1.017 | 1.003 - 1.032 | 0.021 | 1.018 | 1.000 - 1.036 | 0.050 |
| Diabetes duration, years | 0.988 | 0.956 - 1.021 | 0.467 | 1.006 | 0.969 - 1.045 | 0.745 | 0.962 | 0.923 - 1.003 | 0.068 |
| DR: Mild | 1.352 | 0.661 - 2.760 | 0.406 | 1.422 | 0.638 - 3.170 | 0.391 | 1.187 | 0.451 - 3.122 | 0.728 |
| Advanced | 2.658 | 1.032 - 6.952 | 0.044 | 1.630 | 0.525 - 5.061 | 0.398 | 4.710 | 1.475 - 15.039 | 0.009 |

CI, confidence interval; DBP, dyastolic blood pressure; PP, pulse pressure; OR, odds ratio. *The logistic model showed a good discrimination with an AUC of 0.883 (95%CI: [0.846, 0.919] and no significant lack of calibration (Hosmer-Lemeshow test p-value= 0.15). **†**The burden of carotid plaques was modeled by a multinomial logistic regression model.

**Table S2**. Multivariate and multinomial logistic regression models of the presence/absence of atherosclerotic plaque, none vs one plaque, and none vs multiple (≥2) plaques in patients with type 1 diabetes with DR as a variable introduced as presence/absence.

|  | **Presence of any carotid plaques vs. No plaque *** | | | **Burden of carotid plaques †** | | | | | |
| --- | --- | --- | --- | --- | --- | --- | --- | --- | --- |
|  |  |  |  | **One plaque vs. No plaque** | | | **Multiple plaques vs. No plaque** | | |
|  | **OR** | **95% CI** | **p** | **OR** | **95% CI** | **p** | **OR** | **95% CI** | **p** |
| Sex, woman | 0.792 | 0.415 - 1.505 | 0.478 | 1.018 | 0.484 - 2.144 | 0.962 | 0.517 | 0.221 - 1.212 | 0.130 |
| Age, years | 1.103 | 1.064 - 1.147 | <0.001 | 1.069 | 1.023 - 1.117 | 0.003 | 1.148 | 1.092 - 1.206 | <0.001 |
| BMI, kg/m^2^ | 1.014 | 0.939 - 1.094 | 0.725 | 1.008 | 0.923 - 1.100 | 0.864 | 1.021 | 0.925 - 1.128 | 0.677 |
| Dyslipemia | 3.288 | 1.768 - 6.198 | <0.001 | 3.827 | 1.791 - 8.177 | 0.001 | 2.952 | 1.309 - 6.658 | 0.010 |
| LDL, mg/dL | 1.006 | 0.995 - 1.018 | 0.311 | 1.002 | 0.989 - 1.015 | 0.765 | 1.013 | 0.998 - 1.028 | 0.092 |
| DBP, mmHg | 0.997 | 0.966 - 1.029 | 0.869 | 1.015 | 0.978 - 1.053 | 0.435 | 0.974 | 0.935 - 1.014 | 0.200 |
| PP, mmHg | 1.030 | 1.007 - 1.054 | 0.010 | 1.017 | 0.990 - 1.044 | 0.216 | 1.048 | 1.018 - 1.079 | 0.002 |
| Smoking, yes | 2.204 | 1.175 - 4.223 | 0.015 | 2.116 | 1.016 - 4.409 | 0.046 | 2.377 | 1.015 - 5.568 | 0.047 |
| Urine alb/creat | 1.020 | 1.007 - 1.037 | 0.008 | 1.018 | 1.003 - 1.034 | 0.018 | 1.022 | 1.004 - 1.040 | 0.015 |
| Diabetes duration, years | 0.991 | 0.960 - 1.024 | 0.596 | 1.007 | 0.970 - 1.045 | 0.734 | 0.970 | 0.932 - 1.010 | 0.138 |
| DR, yes | 1.638 | 0.851 - 3.167 | 0.140 | 1.445 | 0.677 - 3.082 | 0.342 | 1.925 | 0.829 - 4.469 | 0.129 |

CI, confidence interval; DBP, dyastolic blood pressure; PP, pulse pressure; OR, odds ratio. *The logistic model showed a good discrimination with an AUC of 0.883 (95%CI: [0.847, 0.919] and no significant lack of calibration (Hosmer-Lemeshow test p-value= 0.29). **†**The burden of carotid plaques was modeled by a multinomial logistic regression model.

**Table S3.** Multivariate and multinomial logistic regression models of the presence/absence of atherosclerotic plaque, none vs one plaque, and none vs multiple (≥2) plaques in the whole study group.

|  | **Presence of carotid plaques**  **vs. No plaque *** | | | **Burden of carotid plaques †** | | | | | |  |
| --- | --- | --- | --- | --- | --- | --- | --- | --- | --- | --- |
|  |  |  |  | **One plaque vs. No plaque** | | | **Multiple vs. No plaque** | | | |
|  | **OR** | **95% CI** | **p** | **OR** | **95% CI** | **p** | **OR** | **95% CI** | **p** | |
| Sex, woman | 0.699 | 0.441 - 1.103 | 0.125 | 0.891 | 0.518 - 1.533 | 0.678 | 0.510 | 0.281 - 0.928 | 0.028 | |
| Age, years | 1.097 | 1.070 - 1.126 | <0.001 | 1.080 | 1.048 - 1.112 | <0.001 | 1.119 | 1.084 - 1.156 | <0.001 | |
| BMI, Kg/m^2^ | 1.055 | 0.999 - 1.114 | 0.052 | 1.049 | 0.985 - 1.118 | 0.138 | 1.059 | 0.989 - 1.135 | 0.103 | |
| Dyslipidemia | 2.879 | 1.765 - 4.720 | <0.001 | 3.250 | 1.812 - 5.830 | <0.001 | 2.492 | 1.333 - 4.656 | 0.004 | |
| LDL cholesterol, mg/dL | 1.007 | 0.999 - 1.014 | 0.099 | 1.005 | 0.996 - 1.014 | 0.299 | 1.009 | 0.999 - 1.020 | 0.069 | |
| DBP, mmHg | 0.999 | 0.976 - 1.023 | 0.950 | 1.009 | 0.981 - 1.037 | 0.542 | 0.988 | 0.959 - 1.018 | 0.448 | |
| PP, mmHg | 1.037 | 1.018 - 1.056 | <0.001 | 1.025 | 1.003 - 1.047 | 0.026 | 1.052 | 1.028 - 1.076 | <0.001 | |
| Interaction diabetes - smoking ‡ |  |  |  |  |  |  |  |  |  | |
| Type 1 diabetes if no smoking | 0.526 | 0.250 - 1.096 | 0.088 | 0.467 | 0.198 - 1.100 | 0.082 | 0.643 | 0.231 - 1.790 | 0.398 | |
| Type 1 diabetes if smoking | 1.378 | 0.720 - 2.649 | 0.333 | 1.701 | 0.765 - 3.781 | 0.193 | 1.117 | 0.496 - 2.512 | 0.789 | |
| Smoking if no type 1 diabetes | 0.972 | 0.501 - 1.893 | 0.933 | 0.671 | 0.300 - 1.498 | 0.330 | 1.573 | 0.635 - 3.897 | 0.328 | |
| Smoking if type 1 diabetes | 2.546 | 1.397 - 4.728 | 0.003 | 2.443 | 1.201 - 4.971 | 0.014 | 2.733 | 1.254 - 5.957 | 0.012 | |

CI, confidence interval; DBP, dyastolic blood pressure; PP, pulse pressure; OR, odds ratio, *The logistic regression model showed a good discrimination with an AUC of 0.865 (95%CI: [0.835, 0.894] and no significant lack of calibration (Hosmer-Lemeshow test p-value= 0.12). **†**The burden of carotid plaques was modeled by a multinomial logistic regression model. ‡OR were estimated from the fitted model by fixing the value of the second variable in case of interaction.

test p-value= 0.12) (1). The burden of carotid plaques was modeled by a multinomial logistic regression model (2).
